# Deciphering molecular details in the assembly of alpha-type carboxysome

**DOI:** 10.1038/s41598-018-33074-x

**Published:** 2018-10-10

**Authors:** Yilan Liu, Xinyuan He, Weiping Lim, Joshua Mueller, Justin Lawrie, Levi Kramer, Jiantao Guo, Wei Niu

**Affiliations:** 1 Department of Chemical & Biomolecular Engineering, University of Nebraska-Lincoln, Lincoln, Nebraska 68588 United States; 2 Department of Chemistry, University of Nebraska-Lincoln, Lincoln, Nebraska 68588 United States

## Abstract

Bacterial microcompartments (BMCs) are promising natural protein structures for applications that require the segregation of certain metabolic functions or molecular species in a defined microenvironment. To understand how endogenous cargos are packaged inside the protein shell is key for using BMCs as nano-scale reactors or delivery vesicles. In this report, we studied the encapsulation of RuBisCO into the *α*-type carboxysome from *Halothiobacillus neapolitan*. Our experimental data revealed that the CsoS2 scaffold proteins engage RuBisCO enzyme through an interaction with the small subunit (CbbS). In addition, the N domain of the large subunit (CbbL) of RuBisCO interacts with all shell proteins that can form the hexamers. The binding affinity between the N domain of CbbL and one of the major shell proteins, CsoS1C, is within the submicromolar range. The absence of the N domain also prevented the encapsulation of the rest of the RuBisCO subunits. Our findings complete the picture of how RuBisCOs are encapsulated into the *α*-type carboxysome and provide insights for future studies and engineering of carboxysome as a protein shell.

## Introduction

Bacterial microcompartments (BMCs) are natural proteinaceous cell organelles that encapsulate functionally relevant enzymes to execute designated steps of biological pathways^1–4^. BMCs serve as a vesicular scaffold for the co-localization and physical separation of pathway enzymes from the rest of the cytosolic contents. The benefits and the biological significances of BMCs are often inferred from the functions of the encapsulated cargos. The most well studied BMCs include carboxysomes that participate in CO_2_ fixations in phototrophic and some chemoautotrophic bacteria^5–7^ and metabolosomes, such as Pdu and Eut BMCs that harbor the 1,2-propanediol and ethanolamine degradation pathways in *Salmonella enterica*^8,9^. Carboxysome plays a major role in the CO_2_-concentrating mechanism (CCM) through its selectively permeable protein shell to enhance the lumen concentration of CO_2_, which is produced by the encapsulated carbonic anhydrase (CA) and further serves as the substrate of the co-localized ribulose-1,5-bisphosphate carboxylase/oxygenase (RuBisCO)^10,11^. The local environment with elevated CO_2_ level accelerates the overall rate of CO_2_ fixation by benefitting bacterial RuBisCOs, which generally have higher *k*_*cat*_ and *K*_m_ values than plant enzymes^12^. In addition to providing kinetic advantages, the protein shells of metabolosome-type of BMCs are considered as the major barrier to limit the free diffusion of cytotoxic pathway intermediates, such as propionaldehyde and acetaldehyde, to the cytosol and therefore ensure the fitness of cells.

Encapsulation by naturally occurring BMCs presents an elegant solution to a general orthogonality problem that is encountered in various formats in cellular engineering, such as how to break the diffusion-controlled kinetic barriers and how to minimize noise in a genetic circuit. BMCs are emerging as a promising type of protein scaffold structure in synthetic biology^13–16^. The practical application of BMCs relies on solving a basic research question: how functional proteins are selectively encapsulated into the protein shell. The answer is key to the introduction of foreign protein cargos into artificial BMC as nano-scale bioreactors or as delivery vesicles^17–21^. Mechanistic studies on protein encapsulation into the Pdu and Eut BMCs of *S. enterica* revealed that the N-termini of functional proteins, such as PduA and EutG, serve as signal peptides to interact with shell proteins and are responsible for the formation of BMCs around the cargos^18,22,23^. A similar mechanism was also uncovered in metabolosomes from other bacterial species, albeit the signal peptide may reside on the C-termini^19,24^. This knowledge guided efforts toward the engineering of artificial interaction motifs for foreign cargo protein encapsulation in BMC^25,26^. Studies on the biogenesis of the *β*-type carboxysome revealed a slightly altered approach for the encapsulation of RuBisCO and other proteins. A separate encapsulation peptide (EP), which is encoded by the *ccmN* gene, forms a tether between the cargo and the shell proteins^27^. Structural comparison revealed a common amphipathic *α*-helix motif that is shared between the signal peptides of metabolosomes and the EP of *β*-type carboxysome^28^. However, regardless of recent progress in understanding the role of *csoS2*-encoded scaffold proteins, CsoS2A and CsoS2B, in the nucleation of RuBisCO assembly, molecular details on how RuBisCO is encapsulated inside the shell of the *α*-type carboxysome are still murky^29^. In particular, sequence analysis of gene clusters of the *α*-carboxysomes failed to identify an EP-like sequence^28^, which indicates a different mechanism of cargo packaging.

The objective of the present work is to understand the encapsulation mechanism of *α*-type carboxysome through the study of interactions between BMC shell/scaffold proteins and cargo proteins. The carboxysome from *Halothiobacillus neapolitan* (ATCC 23641) was used as a model system in this study. The *Halothiobacillus neapolitan* encodes the form 1A/*α*-RuBisCO, which consists of eight large (CbbL, 52.6 kDa) and eight small (CbbS, 15.8 kDa) subunits (L_8_S_8_, PDB: 1SVD). The core of the enzyme is made up of four functional dimers of CbbL arranged around a 4-fold axis, and capped at each end by four noncatalytic CbbS proteins. The CbbL is built up of an N domain and a C domain, which harbors the major structure of the active site. A few residues of the N domain from the adjacent subunit of the dimer complete the substrate binding cavity^30^. Intrigued by the encapsulation function of N-terminal sequences of metabolosome enzymes, we seek to investigate whether the N-terminal domain of the large subunit participates in the encapsulation of RuBisCO into the *α*-type carboxysome. In this study, we present experimental evidences to demonstrate that the N domain of CbbL is essential for the encapsulation of the enzyme. Its interaction with the major shell protein has an apparent *K*_d_ in the submicromolar range. *In silico* docking experiments reveal that a short *α*-helix structural motif embedded in the middle of the N-domain may play a key role in the interaction. Our data also show that the previously identified interaction between scaffold protein CsoS2 and RuBisCO is engaged through the RuBisCO small subunit, CbbS. Overall, this study reveals new details of protein-protein interaction in the process of RuBisCO encapsulation into the *α*-type carboxysome and provides insights for future studies and applications of BMCs.

## Results and Discussion

### Encapsulation of wild-type CbbL into the heterologously expressed carboxysome

**Figure 1:**
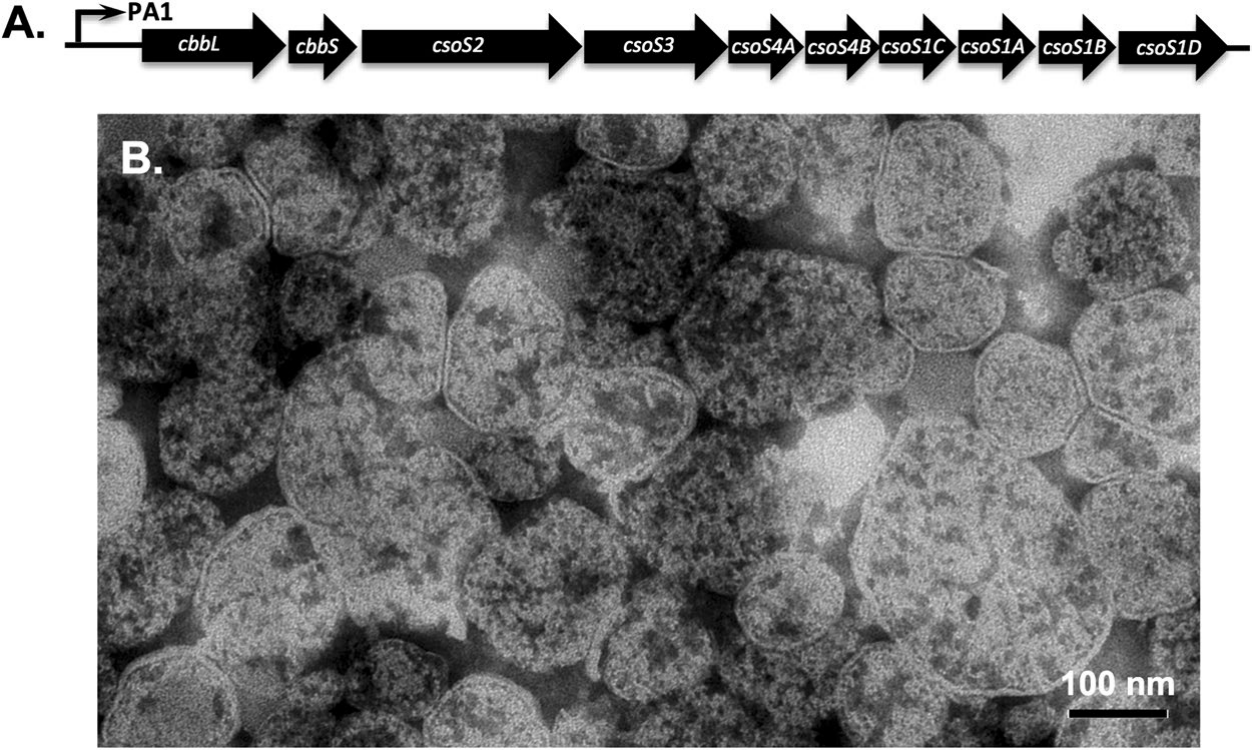
Expression and purification of *H. neapolitan* carboxysome. (**A**) The *cso* gene cluster in plasmid pZS-23641; (**B**) Electron microscopy image of wild-type carboxysome purified from *E. coli* cells transformed with pZS-23641.

**Figure 2:**
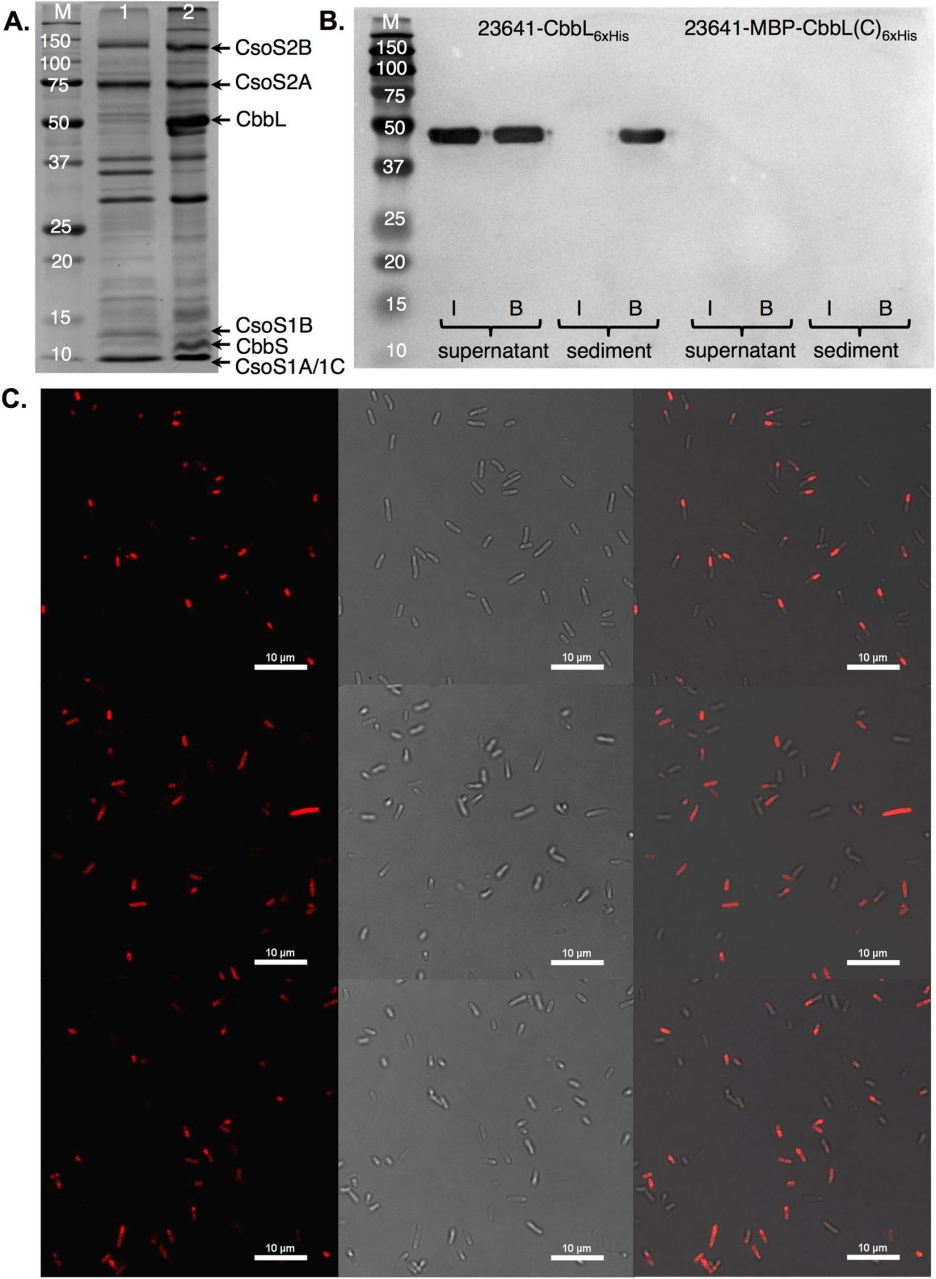
Encapsulation of full-length CbbL and domains of CbbL. (**A**) SDS-PAGE analysis of purified carboxysomes (original image, Fig. S5). M, protein size markers; lane 1, from cells transformed with plasmid pZS-23641-MBP-CbbL(C)_6xHis_; lane 2, from cells transformed with pZS-23641; (**B**) Immunoprecipitation of purified carboxysome (original image, Fig. S6). I, samples from intact carboxysome; B, samples from broken carboxysome; (**C**) Confocal fluorescent microscope analysis of *E. coli* cells. Top panel are cells transformed with pZS-23641-CbbL-mCherry. Middle panel are cells transformed with pZS-23641-CbbL(C)-mCherry. Bottom panel are cells transformed with pZS-23641-CbbL(N)-mCherry. For each panel: left, fluorescent image; center, white-field image; right, overlap image.

We first established a technical platform for the studies, including the expression, isolation, and detection of carboxysome, shell proteins, and/or cargo proteins. The *cso* gene cluster together with the *csoS1D* gene from *H. neapolitan* (ATCC 23641) was cloned behind an isopropyl *β*-D-1-thiogalactopyranoside (IPTG)-inducible promoter on a pSC101-based vector to afford plasmid pZS-23641 (Fig. 1A)^31^. *E. coli* cells transformed with this plasmid were cultured at room temperature in media containing IPTG. Lysate from the harvested cells was subjected to a series of centrifugation steps to obtain fractions of different sedimentation coefficients. The formation of carboxysomes was confirmed by transmission electron microscopy (TEM) analysis of purified fraction (Fig. 1B). The average size of purified carboxysome was around 100 nm, which is consistent with a previous report^32^. Majority of the purified carboxysome had clear and continuous boundaries, which indicates intact physical structures. A small percentage of the sample appeared to be broken presumably due to the lengthy experimental procedure. SDS-PAGE analysis showed protein bands that correlate to the sizes of major shell proteins (CsoS1A/1C, CsoS1B) and encapsulated proteins (CbbL, CbbS, CsoS2A, and CsoS2B) in purified carboxysome (Fig. 2A, lane 2). Bands of minor shell proteins (CsoS1D, CsoS4A, and CsoS4B) and encapsulated carbonic anhydrase (CsoS3) could not be unambiguously identified, which is consistent with a previous report^32^.

To facilitate the encapsulation study, plasmid pZS-23641-CbbL_6xHis_ was constructed to install a six-histidine tag to the C terminus of the CbbL protein encoded by the *cso* cluster. Modified carboxysomes were purified from *E. coli* cells and subjected to immunoprecipitation experiments^22^. Samples of intact and broken carboxysome were incubated with anti-6xHis antibody, which was subsequently precipitated by protein A agarose at low centrifugation speed. Obtained supernatant fractions of the two samples contained either the intact carboxysome or proteins of broken carboxysome. Released proteins from the agarose beads were analyzed as the precipitate fractions. Western blots using the anti-6xHis antibody detected the CbbL-6xHis protein in supernatant fractions from both intact and broken carboxysome samples, which confirmed that the protein was expressed from the modified *cso* gene cluster (Fig. 2B). Furthermore, the CbbL-6xHis protein was only detected in the precipitate fraction of the broken carboxysome, but not in the sample of the intact carboxysome (Fig. 2B). These observations support the conclusion that the CbbL-6xHis protein was inaccessible to anti-6xHis antibody when it was encapsulated into the intact protein shell of carboxysome.

### N domain of CbbL (CbbL(N)) is essential for encapsulation

We hypothesized that the N domain of CbbL is necessary for its encapsulation into functional carboxysome. To this end, we initially sought to apply the same immunoprecipitation method to examining the encapsulation of CbbL protein that lacks the N domain (a.a. 1–136). However, protein expression analysis showed that the C domain of CbbL (CbbL(C), a.a. 137–473) was mainly expressed as inclusion bodies (Fig. S1A). We were unable to separate the inclusion bodies from the carboxysome fraction using sucrose gradient ultracentrifugation method. As a result, the C domain of CbbL was detected in both the broken and the intact fraction of the purified carboxysome in the immunoprecipitation experiment, which complicated the interpretation of the results (Fig. S1B). To solve the problem, we improved the solubility of the C domain by fusing it to the C-terminus of the maltose binding protein (MBP) (Fig. S1C). Plasmid was then constructed by replacing CbbL_6xHis_ with MBP-CbbL(C)_6xHis_ gene. Carboxysome was purified from *E. coli* strain transformed with plasmid pZS-23641-MBP-CbbL(C)_6xHis_. In comparison to the SDS-PAGE of wild-type carboxysome derived from pZS-23641, protein bands of major shell proteins and encapsulated proteins (CsoS2A and CsoS2B) derived from pZS-23641-MBP-CbbL(C)_6xHis_ were still visible (Fig. 2A, lane 1). However, a band with the size of MBP-CbbL(C)_6xHis_ could not be unambiguously identified. Immunoprecipitation experiment to detect the MBP-CbbL(C)_6xHis_ protein in the purified carboxysome also showed negative result (Fig. 2B). These data indicated that the C domain of CbbL was not encapsulated in the carboxysome. Close inspection of SDS-PAGE of the wild-type (Fig. 2A, lane 2) and 23641-MBP-CbbL(C)_6xHis_ carboxysomes (Fig. 2A, lane 1) also revealed a significantly reduced presence of the small subunit of RuBisCO, CbbS, when the N domain of CbbL was not expressed.

As it is possible that the MBP itself and not the lack of the N domain prevented the encapsulation of the MBP-CbbL(C)_6xHis_ fusion protein, we designed and conducted another set of experiments. In these experiments, a red fluorescent protein (mCherry) was fused either to the intact or to the C domain of CbbL. The fluorescence of *E. coli* strains transformed with pZS-23641-CbbL-mCherry or pZS-23641-CbbL(C)-mCherry was studied under confocal microscope (Fig. 2C). Fluorescence foci formation was clearly observed in cells expressing CbbL-mCherry (Fig. 2C, top panel), which is a sign of localization in the cytosol due to the encapsulation by the carboxysome^32^. On the other hand, evenly dispersed fluorescent signals were observed in cells expressing the CbbL(C)-mCherry fusion protein (Fig. 2C, middle panel). Above data suggest that the N domain of CbbL is essential for its packaging into the carboxysome.

**Figure 3:**
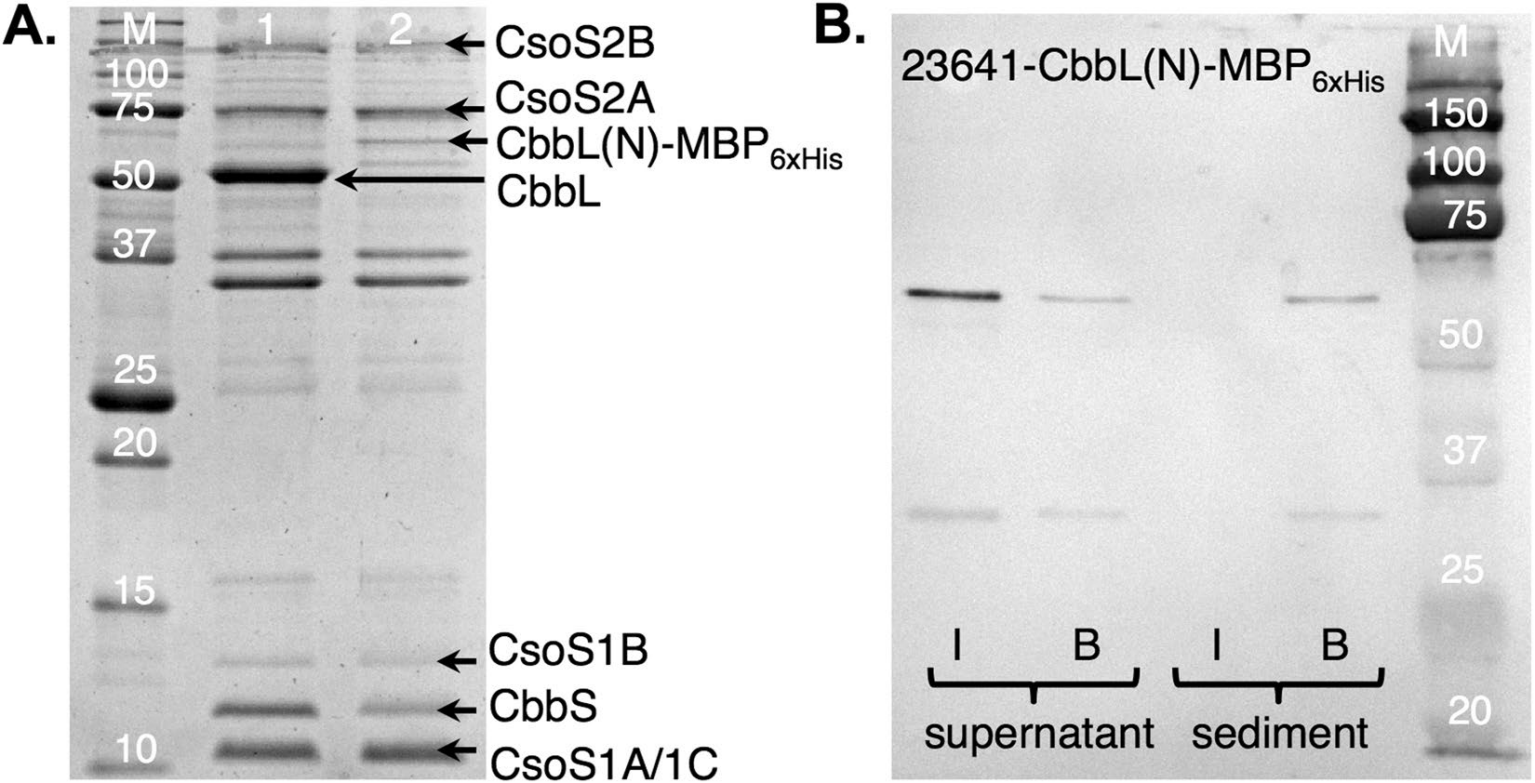
N domain of CbbL directs foreign proteins to carboxysome. (**A**) SDS-PAGE analysis of purified carboxysomes (original image, Fig. S7). M, protein size markers; lane 1, from cells transformed with plasmid pZS-23641-CbbL_6xHis_; lane 2, from cells transformed with pZS-23641-CbbL(N)-MBP_6xHis_; (**B**) Immunoprecipitation of purified carboxysome (original image, Fig. S8). I, samples from intact carboxysome; B, samples from broken carboxysome.

To further investigate whether the N domain of CbbL is capable of localizing foreign proteins to carboxysome, we fused either MBP_6xHis_ or mCherry_6xHis_ to the C-terminus of the domain. Carboxysome was purified from *E. coli* cells transformed with pZS-23641-CbbL(N)-MBP_6xHis_ (Fig. 3A, lane 2). In comparison with purified wild-type carboxysome (Fig. 3A, lane 1), a new protein band corresponding to the size of CbbL(N)-MBP_6xHis_ fusion protein was observed. Western blot experiment confirmed the protein band contained His tag (Fig. S2B). The intensity of this band is significantly lower than the band of CbbL in the wild-type carboxysome, although the two samples of purified carboxysome contained similar amounts of shell proteins (CsoS1A/1B/1C) and scaffold proteins (CsoS2A/2B) (Fig. 3). The purified carboxysome was then subjected to immunoprecipitation experiment (Fig. 3B), which showed that the CbbL(N)-MBP_6xHis_ fusion protein was located to the purified carboxysome (Fig. 3B). We further studied the distribution of mCherry protein in *E. coli* cells expressing pZS-23641-CbbL(N)-mCherry (Fig. 2C, bottom panel). Significant heterogeneity in fluorescence distribution was observed within the cell population. Analysis showed that around 35% of the fluorescence signal formed foci in 23641-CbbL(N)-mCherry sample, while 100% of the signals formed foci in 23641-CbbL-mCherry sample and less than 5% in 23641-CbbL(C)-mCherry sample. Above data support the conclusion that the N domain of CbbL is capable of directing foreign protein to carboxysome, although the efficiency is low due to apparent stochastic effect. The expression of the N domain of CbbL also largely recovered the encapsulation of small subunit of RuBisCO, CbbS (Fig. 3A).

### Scaffold protein CsoS2 interacts with CbbS

**Figure 4:**
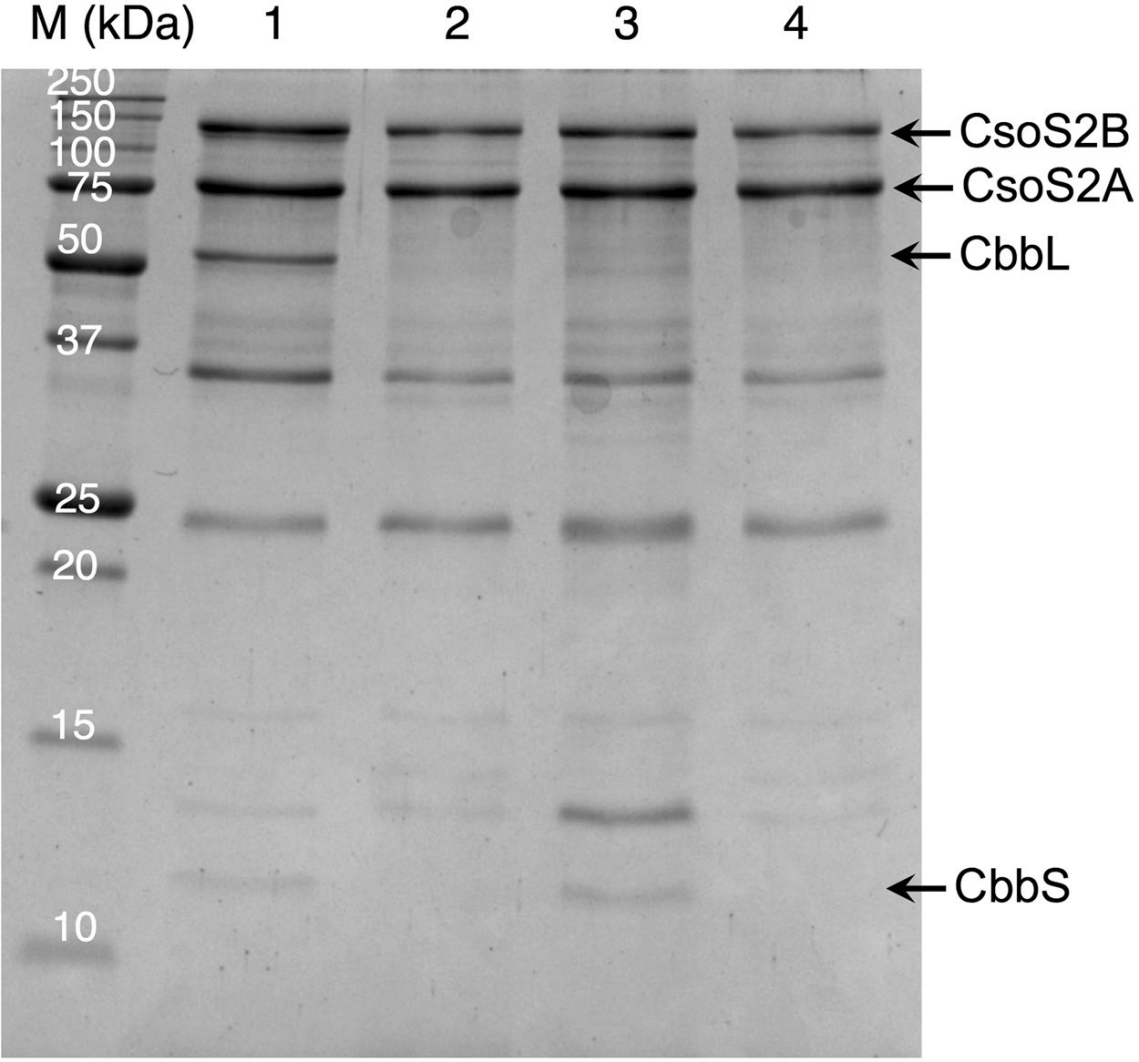
Pull-down assay between 6xHis-CsoS2s and CbbL/CbbS. In each lane, the following carboxysome-related proteins were co-expressed with 6xHis-CsoS2s: lane 1, CbbL and CbbS; lane 2, CbbL; lane 3, CbbS; lane 4, none. M, protein size markers. Original image is provided as Fig. S9 in Supplemental Information.

To understand how the N domain of CbbL participates in the encapsulation process, we first examined its interaction with CsoS2 protein. It is known that CsoS2 is absolutely conserved in organisms that express *α*-carboxysomes. Recent studies demonstrated that this structurally flexible protein interacts with both major shell proteins and RuBisCO^29^. It was further hypothesized that CsoS2 serves as a scaffold to recruit shell proteins and RuBisCO prior to the assembly of carboxysome^29^. However details on the interaction between CsoS2 and RuBisCO have not been studied. To probe whether the N domain of CbbL plays any role in this process, we conducted pull-down assays using cells that co-expressed different combinations of CsoS2 (with N-6xHis tag) and CbbL/CbbS proteins (Fig. 4). When CsoS2 and CbbS were co-expressed in *E. coli* cells, the fraction purified using immobilized Ni resin contained both CsoS2 and CbbS (Fig. 4, lane 3). On the other hand, the co-purification was not observed for CbbL when it was expressed together with CsoS2 (Fig. 4, lane 2). Furthermore, when the RuBisCO complex, including both CbbL and CbbS, was co-expressed with CsoS2, CbbL was also observed in the pull-down fraction (Fig. 4, lane 1). The results support the notion that scaffold CsoS2 recruits RuBisCO through its interaction with the small subunit (CbbS) of the RuBisCO, which eliminates the possibility that the role of CbbL(N) in RuBisCO encapsulation is through its interaction with CsoS2 proteins.

### The N domain of CbbL (CbbL(N)) interacts with major shell proteins

The lack of direct interaction between CsoS2 proteins and CbbL(N) promoted us to investigate whether the N domain of CbbL interacts with any shell proteins of carboxysome. To co-express CbbL(N) and shell proteins in *E. coli* for pull-down assays, two plasmids were constructed. The first plasmid encodes a fusion protein of CbbL(N) and mCherry with a C-terminal 6xHis tag. The fluorescence protein was fused to the C-terminus of CbbL(N) and used as a visual signal to facilitate the protein purification. All genes that encode the *H. neapolitan* shell proteins, including CsoS1A/1B/1C/1D and CsoS4A/4B, were cloned into a second plasmid, pZS-23641-CsoS41. Above two plasmids were co-transformed into *E. coli* for protein expression and the cell extract was subjected to the pull-down assay. By comparing to a control sample of the CbbL(N)-mCherry-6xHis protein that was purified separately in parallel, an additional band was unambiguously identified in the SDS-PAGE of the pull-down sample (Fig. S3). Proteomics analysis confirmed that the identified band consisted of CsoS1A and CsoS1C with 91% sequence coverage for each protein. Since the two shell proteins are of the same length (98 a.a.) and differ only in two amino acid residues, they could not be resolved in our SDS-PAGE experiments.

**Figure 5:**
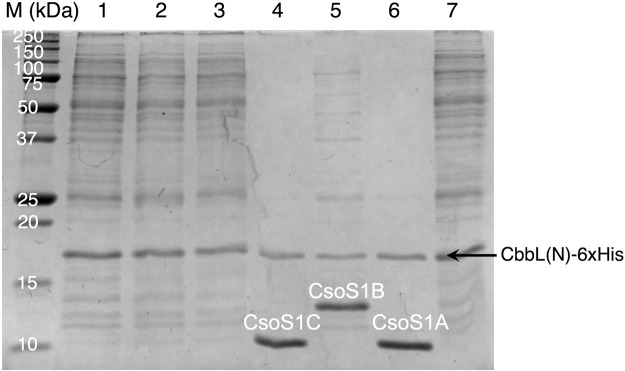
Pull-down assay between CbbL(N)-6xHis and shell proteins of *H. neapolitan* carboxysome. In addition to CbbL(N)-6xHis, cells also expressed the following carboxysome-related proteins: lane 1, CsoS4B; lane 2. CsoS4A; lane 3. CsoS1D; lane 4. CsoS1C; lane 5. CsoS1B; lane 6. CsoS1A; lane 7. none. M, protein size markers. Original image is provided as Fig. S10 in Supplemental Information.

To verify our above observations and to exclude the possibility that interactions between CsoS1A/1C and mCherry led to the pull-down effect, we generated a control construct in which the 6xHis tag was fused directly to the C terminus of CbbL(N). Plasmids encoding individual shell protein were also constructed. Another round of pull-down assays were conducted between Cbb(N)-6xHis and each shell protein separately. The results confirmed that the N domain of CbbL interacts with shell proteins CsoS1A and CsoS1C (Fig. 5). In addition, interaction between CbbL(N) and CsoS1B was also detected (Fig. 5). On the other hand, no pull-down effect was observed for CsoS1D, CsoS4A, and CsoS4B (Fig. 5). It was estimated in previous reports that CsoS1A/1B/1C (Pfam00936, BMC-H)^33,34^ exist in 3510 copies per carboxysome, while the copy numbers of CsoS1D (BMC-T) and CsoS4A/4B (Pfam 03319, BMC-P) were too low to be determined in wild-type carboxysomes purified from *H. Neapolitan*^35^. Interactions between the N domain of CbbL and the three major shell proteins indicate possible role that is played by CbbL(N) during the encapsulation of RuBisCO.

### Structural motifs of CbbL(N) that is responsible for the interaction

**Figure 6:**
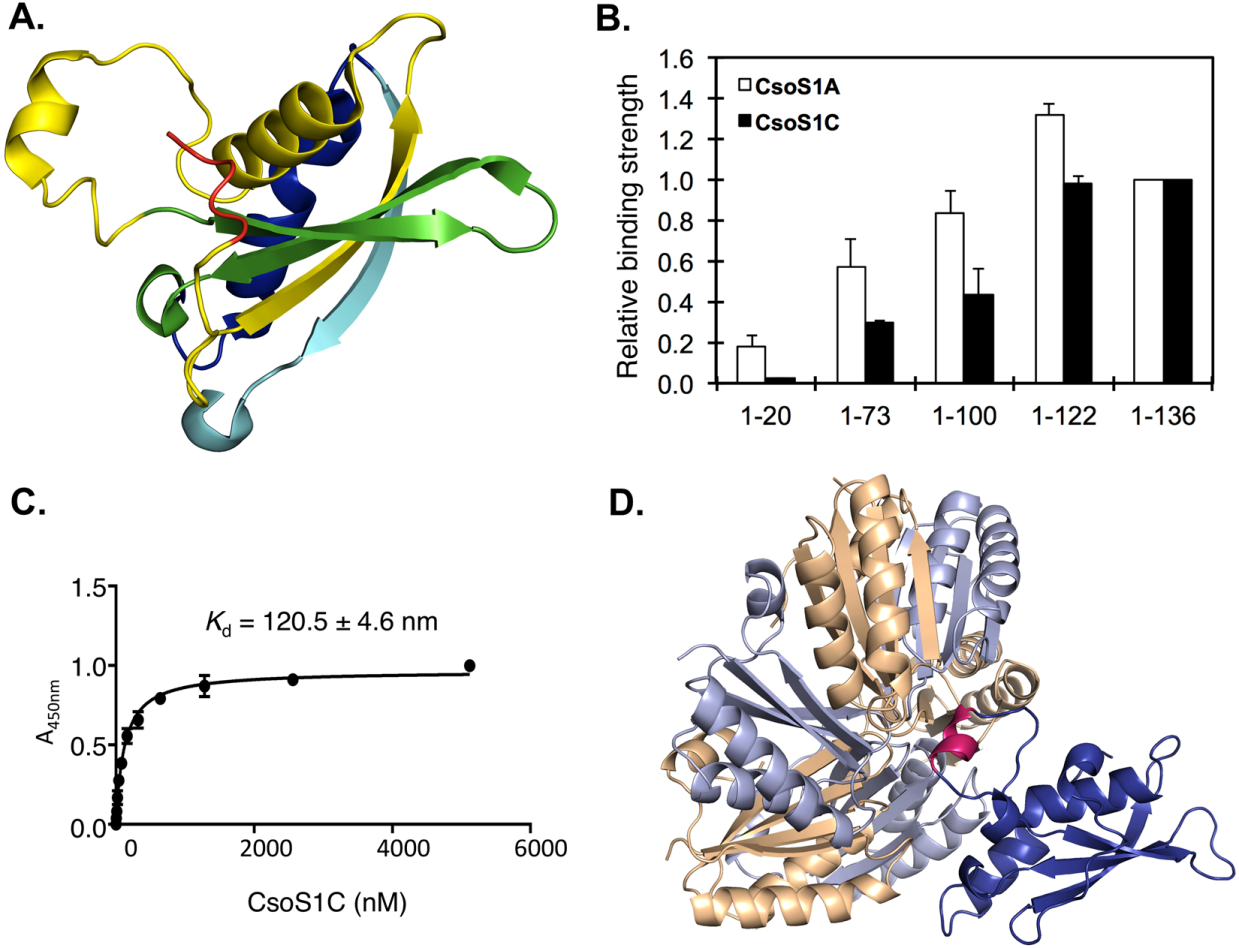
Interaction of truncated CbbL(N) with major shell proteins of *H. neapolitan* carboxysome. (**A**) Structure of CbbL(N) colored according to the truncated segments; (**B**) The relative binding strength between segments of CbbL(N) and shell proteins; (**C**) The binding affinity assay between CsoS1C and CbbL(1–136); (**D**) *In silico* simulation of interaction between hexamer of CsoS1C and CbbL(1–136). The hexamer of CsoS1C is colored in gold and purple. CbbL(N) is colored in blue, while the short *α* helix (a.a. 63–67) is colored in pink.

To investigate whether the entire N domain (a.a. 1–136) of CbbL contribute to the interaction with major shell proteins, we expressed five truncated segments of CbbL(N) based on its secondary structural units (Fig. 6A). Segment 1–20 represented the flexible N terminus (red, Fig. 6A) that was mainly ‘invisible’ in the X-ray structure. Segment 1–73 contained one of the central *β* strand followed by one large and one short *α* helices (yellow, Fig. 6A). Segment 1–100 covered two additional central *β* strands (green, Fig. 6A), while segment 1–122 covered the last *α* helix (blue, Fig. 6A) and 1–136 included an additional *β* strand (cyan, Fig. 6A). Each segment was fused to the N terminus of MBP that contained a C-terminal 6xHis tag. The interaction between truncated CbbL(N) with major shell proteins CsoS1A and CsoS1C was studied using pull-down assays (Fig. S4). The ligand binding ratio between the CbbL(N) segment and the shell protein was calculated using signals from densitometry measurement in the assays. The relative binding strength of a truncated segment was expressed as the percentage of its ligand-binding ratio to that of the entire N domain (Fig. 6B). Data indicated that the unstructured N-terminal peptide that contains the first 20 amino acids played insignificant role in the binding of CbbL(N) with the shell proteins, which is different from the observations of signal peptides in functional proteins of the Pdu and Eut metabolosomes. For CsoS1A and CsoS1C, the relative binding strength with truncated N domain continuously increased and reached maxima when CbbL(1–122) was used. Only a small difference in binding strength was observed between CbbL(1–122) and CbbL(N).

Due to the high sequence homology between CsoS1A and CsoS1C and their similar behaviors in the pull-down assay against truncated CbbL(N) protein, further studies on the interaction between the N domain of CbbL and major shell proteins focused on CsoS1C. The binding affinity between CbbL(N) and CsoS1C was examined by semi-quantitative ELISA experiments^36^. In brief, wells of microtiter plates were coated with MBP tagged CbbL(1–136), into which different concentrations of the CsoS1C-6xHis protein were applied. Following removal of the unbound proteins by washing, the amount of CsoS1C that bound to CbbL(1–136) was quantified by immunoassays. The interaction between CbbL(1–136) and CsoS1C showed a strong binding affinity with an apparent *K*_d_ of 120.5 ± 4.6 nM.

*In silico* docking experiment was subsequently conducted to simulate the possible mode of interaction between CbbL(N) and CsoS1C. The hexameric structure of CsoS1C (PDB: 3H8Y)^37^ and the CbbL (PDB: 1SVD) were used in rigid body docking in the ZDOCK module of Discovery Studio suite. The top three conformations with the lowest energy all showed possible interaction between a short *α* helix formed by residues 63 to 67 of CbbL(N) and the convex side of the CsoS1C hexamer (Fig. 6D). The simulation result supported observations in CbbL(N) truncation experiments where significant interactions were first observed when fragment 1–73 was examined. The identified short *α* helix is held between *α* helix (a.a. 42–54) and *β* sheet (a.a. 76–82), which indicates that additional structural units may further constraint the short *α* helix for optimal interaction with the shell proteins.

## Conclusion

BMC is a promising type of protein scaffold for applications in synthetic biology, drug delivery and material science. Understanding the assembly mechanism of BMCs is critical for further engineering efforts. This research focused on studying the interactions between subunits of RuBisCO and shell/scaffold proteins of the *α*-type carboxysome from *H. neapolitan*. Our data showed that the small subunit (CbbS) of RuBisCO interacts with the scaffold proteins (CsoS2), while the N domain of the large subunit (CbbL(N)) of RuBisCO interacts with all major shell proteins CsoS1A, 1B and 1C with submicromolar binding affinity. Computer simulation revealed a possible mode of interaction between the CbbL(N) domain and shell proteins. The findings reveal molecular details of protein-protein interactions in the process of *α*-type carboxysome assembly and further support previous hypothesis of simultaneous assembly of the shell and encapsulation of RuBisCO enzymes^38^. In addition to completing the picture of *α*-type carboxysome assembly, findings from this work also provide insights for future engineering of carboxysome for biotechnological applications.

## Methods

### Material and general methods

Primers (Table S1) were ordered from Sigma. Plasmids (Table S2) were constructed using standard molecular biology techniques^39^. DNA sequencing services were provided by Eurofins MWG Operon. Restriction enzymes, Antarctic phosphatase (AP) and T4 DNA ligase were purchased from New England Biolabs. KOD hot start DNA polymerase was purchased from EMD Millipore. All solutions were prepared in deionized water that was further treated by Barnstead Nanopure® ultrapure water purification system (Thermo Fisher Scientific). LB medium (1 L) contained Bacto tryptone (10 g), Bacto yeast extract (5 g), and NaCl (10 g). Antibiotics were added where appropriate to following final concentrations: ampicillin, 100 mg L^−1^, chloramphenicol, 34 mg L^−1^, kanamycin, 50 mg L^−1^.

### Expression and purification of carboxysome

Plasmid encoding the wild-type or a modified carboxysome was transformed into *E. coli* NEB^®^5-alpha. Cells were cultured in LB media with ampicillin at 37 °C until the OD_600nm_ reached 0.6. Following the addition of IPTG to a final concentration of 0.2 mM, expression of carboxysome was carried out at room temperature for 14 h. Cells were collected by centrifugation, then resuspended in TEMB buffer (5 mM Tris-HCl, pH 8.0, 1 mM EDTA, 10 mM MgCl_2_, 20 mM NaHCO_3_). Purification of carboxysome followed a reported protocol^40^. Obtained pellet of carboxysome was first washed with ultrapure water, then resuspended in TEMB.

### Pull-down assays

Two plasmids, each encoded one protein of interest, were co-transformed into *E. coli* BL21(DE3). Transformed cells were cultured in LB medium with appropriate antibiotics at 37 °C until OD_600_ reached 0.6. Protein expression was induced with IPTG at 0.4 mM and carried out at 30 °C for 12 h. The cells were harvested by centrifugation at 5,000 *g* for 10 min. Collected cells were suspended in TEMB with 200 mM NaCl, then lysed by sonication. Following removal of cell debris by centrifugation at 16,000 *g* for 20 min, the supernatant was applied to a Ni Sepharose 6 Fast Flow (GE Healthcare) column. The column was eluted stepwise with five column volumes of buffers containing 5 mM, 50 mM, then 250 mM of imidazole. All elution fractions were analyzed by SDS-PAGE to test for co-elution of proteins with and without the 6xHis tag.

### Immunoprecipitation

Protein A agarose beads (Pierce^TM^, ThermoFisher Scientific) were first washed with PBS buffer, then diluted tenfold in PBS prior to mixing with 6xHis tag monoclonal antibody (Invitrogen^TM^, ThermoFisher Scientific). The bead suspension was incubated at 4 °C for 1 h with gently shaking. Purified carboxysome was first treated with Ni Sepharose 6 Fast Flow resin to remove His-tagged protein that was leaked out of the carboxysome during the sample preparation process. To obtain broken carboxysome, purified sample was subjected to sonication using Model 120 sonic dismembrator (Fisherbrand^TM^) at 50% amplitude for 2 min. Samples of broken or intact carboxysome were mixed with antibody-treated protein A beads. Following incubation at 4 °C for 1 h with gentle shaking, the beads were collected by centrifugation at 2,500 *g* for 2 min. The supernatant was saved as the supernatant fractions for further analysis. The beads were washed three times with 0.5 mL of IP buffer (25 mM Tris-HCl, pH 7.2, 150 mM NaCl). To elute the protein, the beads were suspended in 50 μL of glycine-HCl buffer (150 mM, pH 2.94) and incubated for 5 minutes. Following centrifugation at 2,500 *g* for 2 min, the supernatant was collected as the sediment fraction from the immunoprecipitation step. The supernatant and sediment fractions were analyzed by Western blots for the presence of 6xHis tag using the 6xHis tag monoclonal antibody together with the goat anti-mouse IgG-HRP conjugate (BioRad) and Opti-4CN detection kit (BioRad).

### Enzyme-linked immunosorbent assay (ELISA)

The semi-quantitative ELISA experiments followed reported protocols with minor modifications^36^. In brief, MaxiSorp 96 well ELISA plates were coated with 100 μL of MBP-tagged CbbL(1–136) proteins (30 nM in 0.1 M Na_2_CO_3_ (pH 9.6)) at 4 °C for 12 h. After removal of the coating solution, blocking buffer (1 mM CaCl_2_ and 1% BSA in TBS buffer) was added and the plates were incubated at room temperature for 1 h. Following three times of washing with washing buffer (1 mM CaCl_2_ and 0.05% Tween 20 in TBS buffer), solutions of CsoS1C-6xHis protein at varied concentrations (5 nM to 5 μM in blocking buffer) were applied to each well. The plates were incubated for 1 h at room temperature, then washed three times to remove unbound protein. Detection of bound protein used the 6xHis tag monoclonal antibody together with the goat anti-mouse IgG-HRP conjugate and the 3, 3′, 5, 5′-tetramethylbenzidine (TMB) substrate. Following the reaction, the absorbance at 450 nm was measured to quantify the activities of HRP. Dissociation constants were calculated by curve fitting with Hill Equation using Prism 7 (Graphpad).

### Proteomics analysis

Gel band sample was washed, then digested with trypsin overnight at 37 °C. Tryptic peptides were extracted from the gel pieces, dried down, and re-dissolved in 25 μL of 2.5% acetonitrile, 0.1% formic acid. The digests were run on a Q-Exactive-HF mass spectrometer (ThermoFisher Scientific) equipped with a U3000 RSLCnano LC system. All MS/MS data were analyzed using Mascot (Matrix Science, v 2.5.1). Mascot was set up to search the databases with a fragment ion mass tolerance of 0.060 Da and a parent ion tolerance of 10 ppm. Modifications of residues, including deamination of asparagine and glutamine, oxidation of methionine and carbamidomethyl of cysteine, were specified as variable modifications. Scaffold (Proteome Software Inc., v 4.7.5) was used to validate MS/MS based peptide and protein identifications. Peptide identifications were accepted if they could be established at greater than 80.0% probability and protein identifications were accepted if they could be established at greater than 99.0% probability and contained at least 2 identified peptides, with a false discovery rate less than 1%.

### Microscopy methods

For transmission electron microscopy analysis, purified carboxysome were first fixed with glutaraldehyde (4% in PBS buffer) for 30 min at room temperature, washed three time with PBS, then adsorbed for 1 min to a carbon-coated grid. The samples were stained with 1.0% uranyl acetate in deionized water. The grids were imaged using 80 kv accelerating voltage using a Hitachi H7500 transmission electron microscope. For imaging using fluorescent microscope, *E. coli* cells transformed with either pZS-23641-CbbL-mCherry or pZS-23641-CbbL(C)-mCherry were cultured, induced for carboxysome expression, then collected by centrifugation at 5,000 *g* for 10 min. Cells were washed three times with PBS buffer, then fixed with paraformaldehyde (4% in PBS buffer) for 30 min at room temperature. Cells were then washed, resuspended in PBS, loaded on a glass slide, then imaged using Nikon A1R-Ti-2 confocal microscope. Texas red channel was excited at 595 nm and imaged at 620 nm. The percentage of localization of fluorescence signal is analyzed using ImageJ^41^.

## Electronic supplementary material

Supplementary Information
