# Supplementary material for: Deciphering molecular details in the assembly of alpha-type carboxysome
Source: Sci Rep. 2018 Oct 10;8:15062. doi: 10.1038/s41598-018-33074-x (PMC6180065; doi:10.1038/s41598-018-33074-x)
Supplement: Supplementary file 1 — Supplementary Information [file 41598_2018_33074_MOESM1_ESM.pdf]

# Deciphering molecular details in the assembly of alpha-type carboxysome

Yilan Liu,[1] Xinyuan He,[1] Weiping Lim,[1] Joshua Mueller,[1] Justin Lawrie,[2] Levi Kramer,[1] Jiantao Guo[2] and Wei Niu[1]*

1. Department of Chemical & Biomolecular Engineering, University of Nebraska-Lincoln, Lincoln, Nebraska, 68588, United States.

2. Department of Chemistry, University of Nebraska-Lincoln, Lincoln, Nebraska, 68588, United States.

*To whom correspondence should be addressed: wniu2@unl.edu

## Table of Contents


**Table S1.** Primers used in the study. Page 3-4

**Table S2.** Strains and plasmids used in the study. Page 5

Construction of plasmids. Page 6-8

**Figure S1.** Solubility study of CbbL(C) and MBP-CbbL(C) using western blot. Page 9

**Figure S2.** Analysis of purified carboxysome 23641-CbbL(N)-$MBP_{6xHis}$ Page 10

**Figure S3.** Identification of shell proteins that interact with CbbL(N). Page 11

**Figure S4.** Pull-down experiments between truncated CbbL(N) and major shell proteins. Page 12

**Figure S5.** Original SDS-PAGE image of Figure 2A Page 13

**Figure S6.** Original western blot image of Figure 2B Page 14

**Figure S7.** Original SDS-PAGE image of Figure 3A Page 15

**Figure S8.** Original western blot image of Figure 3B Page 16

**Figure S9.** Original SDS-PAGE image of Figure 4 Page 17

**Figure S10.** Original SDS-PAGE image of Figure 5 Page 18

**Figure S11.** Original western blot image of Figure S1A Page 19

**Figure S12.** Original western blot image of Figure S1B Page 20

**Figure S13.** Original western blot image of Figure S1C Page 21

**Figure S14.** Original SDS-PAGE image of Figure S2A Page 22

**Figure S15.** Original SDS-PAGE image of Figure S2B Page 23

**Figure S16.** Original SDS-PAGE image of Figure S3 Page 24

**Figure S17.** Original SDS-PAGE image of Figure S4A Page 25

**Figure S18.** Original SDS-PAGE image of Figure S4B Page 26

**Table S1. Primers used in the study.**

| Primers | Sequences (5' -> 3') (restriction sites underlined) |
|---|---|
| 23641-forward | ttaatctagaggatcctgcgcgaatcccccatcctttcag |
| 23641-reverse | tgcatctagacattagctattcagatttgcgatac |
| csoS1D-forward | aatgtctagatcagggaagatgcgcatgaacaacattg |
| csoS1D-reverse | taggaagctttctagattagaacccttcagcgcgacgcgcac |
| cbbL(C)-His-F | tgatccctcgtaccacac |
| cbbL(C)-His-R | tggtacgagggatcagtggtggtggtggtggtgacgattttgagtgtcgagtttg |
| MBP-cbbL-F | cgggatcctgcgcgaatcccccatcctttcaggaggaactcatgaaaactgaagaaggtaaactgg |
| MBP-cbbL-R | cgtgcggtgggccgccacaggttttaacgtactcgagccttccctcg |
| cbbL-MBP-F | tacgttaaaacctgtggcgg |
| cbbL-MBP-R | ttgatccgccatttggtaat |
| cbbL-mCherry-P1 | acgcgtcgacaaactcgacactcaaaatcgtgtgagcaagggcgaggag |
| cbbL-mCherry-P2 | aagcttcagcttcctttcgggcttt |
| cbbL-mCherry-P3 | aaagcccgaaaggaagctgaagctttgatccctcgtaccacac |
| cbbL-mCherry-P4 | ttgatccgccatttggtaat |
| cbbL-Ndeletion-F | tacgttaaaacctgtggcgg |
| cbbL-Ndeletion-R | catgagttcctcctgaaagg |
| csoS2-F | acgcgtcgacatgcaccaccaccaccaccacccttcacagtcaggaatgaatc |
| csoS2-R | cccaagcttttacttaatcaaccgcgcg |
| cbbL-forward | cctataaaaataggcgtatca |
| cbbL-reverse | ccaagctttctagatcaacgattttgagtgtcgag |
| cbbS-forward | ggaattccatatggctgaaatgcaggattac |
| cbbS-reverse | ccgctcgagttagttgccgcggtagac |
| cbbL(N)-mCherry | gccgcgcggcaccaggccgctgctggcgagtgggaagcgaa |
| mCherry-forward | agcagcggcctggtg |
| mCherry-reverse | gaaagcttcagcttcctttcgggcttt |
| cso41-forward | atgcaagttgagaaaacgttgg |
| cso41-reverse | gagttcctcctgaaaggatggg |
| cbbL(N)-forward | gctctagaattcaattaagctagaggatcctgc |
| cbbL(N)-reverse | cccaagcttcagccggatctcagtg |
| csoS1A-forward | ggaattccatatggctgatgtaactggtattg |
| csoS1A-reverse | ccgctcgagctaggcttgtggcgcctt |
| csoS1B-forward | ggaattccatatggcaacgactcacgg |
| csoS1B-reverse | ccgctcgagttagctattcagatttgcgatacac |

**Table S1. Primers used in the study (cont.).**

| Primers | Sequences (5' -> 3') (restriction sites underlined) |
|---|---|
| csoS1C-forward | ggaattccatatggcagcagtaacagg |
| csoS1C-reverse | ccgctcgagttaagcttcaggggctttcg |
| csoS1D-forward | ggaattccatatgaacaacattgatttgcgc |
| csoS1D-reverse | ccgctcgagttagaacccttcagcgcg |
| csoS4A-forward | ggaattccatatgcaagttgagaaaacgttg |
| csoS4A-reverse | ccgctcgagttactcaccattccactgatc |
| csoS4B-forward | ggaattccatatgcgcgttcgttcc |
| csoS4B-reverse | ccgctcgagtcaagttacccagtgatcgatg |
| cbbL20-reverse | gccgcgcggcaccaggccgctgctcggcatccaataggtctg |
| cbbL73-reverse | gccgcgcggcaccaggccgctgctgtagtagtccatgtcggtcag |
| cbbL100-reverse | gccgcgcggcaccaggccgctgctcaggtcgattgggtaggc |
| cbbL122-reverse | gccgcgcggcaccaggccgctgctcgctttgaagccgaacac |
| cbbL136-reverse | gccgcgcggcaccaggccgctgctggcgagtgggaagcgaa |
| MBP-forward | agcagcggcctggtgccgcgcggcagcggtaaaactgaagaaggtaaactgg |
| MBP-reverse | ccgctcgagccttccctcgatcccg |
| cbbL136-MBPnoHis | ccgctcgagctaccttccctcgatcccg |
| csoS1C6xHis-F | gctctagagattttgaatgagtctttattgagg |
| csoS1C6xHis-R | gactcgagagcttcaggggctttcg |

**Table S2. Strains and plasmids used in the study.**

| Strain or plasmid | Relevant properties | Source/Reference |
|---|---|---|
| **Strains** | | |
| *Halothiobacillus neapolitan* (ATCC 23641) | source of genes | ATCC |
| *E. coli* NEB®5-alpha | cloning host strain | New England Biolab |
| *E. coli* BL21(DE3) | inducible T7 RNA polymerase | Novagen |
| **Plasmids** | | |
| pET30b | cloning vector, $Kan^R$ | Novagen |
| pZS13 | cloning vector, PA1 promoter, SC101 *ori*, $Amp^R$ | Expressys |
| pZA33 | cloning vector, PA1 promoter, p15A *ori*, $Cm^R$ | Expressys |
| pZS-23641 | 8.4 kb carboxysome operon of *H. neapolitan* cloned into pZS13S | this study |
| pZS-23641-CbbL6xHis | 6xHis tag was added to the C terminus of CbbL in pZS23641 | this study |
| pZS-23641-MBP-CbbL(C)6xHis | MBP replaced the N domain of CbbL in pZS-23641-CbbL6xHis | this study |
| pZS-23641-CbbL-mCherry | mCherry was fused to the C terminus of CbbL in pZS23641 | this study |
| pZS-23641-CbbL(C)-mCherry | N domain of CbbL was deleted from pZS-23641-CbbL-mCherry | this study |
| pZS-CsoS2(N6xHis) | CsoS2 expressed with N-terminal 6xHis tag | this study |
| pZA-CbbL | for co-expression of CbbL together with CsoS2(N6xHis) | this study |
| pZA-CbbS | for co-expression of CbbS together with CsoS2(N6xHis) | this study |
| pZA-CbbLS | for co-expression of CbbLS together with CsoS2(N6xHis) | this study |
| pZA-CbbL(N)-mCherry6xHis | N domain of CbbL is fused to mCherry-6xHis through a GSSGLV linker | this study |
| pZS-23641-CsoS41 | encodes all the shell proteins of *H. neapolitan* carboxysome | this study |
| pZA-cbbL(N)6xHis | N domain of CbbL was expressed with a N-terminal 6xHis tag | this study |
| pET-csoS1A | for co-expression of CsoS1A together with CbbL(N)6xHis | this study |
| pET-csoS1B | for co-expression of CsoS1B together with CbbL(N)6xHis | this study |
| pET-csoS1C | for co-expression of CsoS1C together with CbbL(N)6xHis | this study |
| pET-csoS1D | for co-expression of CsoS1D together with CbbL(N)6xHis | this study |
| pET-csoS4A | for co-expression of CsoS4A together with CbbL(N)6xHis | this study |
| pET-csoS4B | for co-expression of CsoS4B together with CbbL(N)6xHis | this study |
| pZA-cbbL(1-20)-MBP6xHis | the 1-20 amino acid segment of CbbL fused to N terminus of MBP6xHis | this study |
| pZA-cbbL(1-73)-MBP6xHis | the 1-73 amino acid segment of CbbL fused to N terminus of MBP6xHis | this study |
| pZA-cbbL(1-100)-MBP6xHis | the 1-100 amino acid segment of CbbL fused to N terminus of MBP6xHis | this study |
| pZA-cbbL(1-122)-MBP6xHis | the 1-122 amino acid segment of CbbL fused to N terminus of MBP6xHis | this study |
| pZA-cbbL(1-136)-MBP6xHis | the N domain of CbbL fused to N terminus of MBP6xHis | this study |
| pZA-cbbL(1-136)-MBP | the N domain of CbbL fused to N terminus of MBP | this study |
| pET-csoS1C6xHis | expression of CsoS1C with C-terminal His tag | this study |

**pZS-23641.** A 7.7 kb DNA fragment that encodes the *cso* operon of *H. neapolitanus* (ATCC 23641) was amplified using primer pair 23641-forward and 23641-reverse. PCR product was cloned between the BamHI and XbaI sites on pZS13 to generate an intermediate plasmid. The *csoS1D* gene of *H. neapolitanus* (ATCC 23641) together with its ribosomal binding site was amplified using primer pair csoS1D-forward and csoS1D-reverse. PCR product was cloned between the XbaI and HindIII sites on the intermediate plasmid to generate pZS-23641.

**pZS-23641-CbbL$_{6xHis}$.** Primer pair cbbL(C)-His-F and cbbL(C)-His-R was used to add an 18 bp sequence, which encodes the Hisx6 tag, to the 3' end of the CbbL gene via reverse PCR using pZS-23641 as the template. The PCR product was subjected to sequence and ligation-independent cloning process to yield pZS-23641-CbbL6xHis.

**pZS-23641-MBP-CbbL(C)$_{6xHis}$.** A 1.1 kb DNA fragment encoding MBP was amplified from plasmid pMAL using primer pair MBP-cbbL-F and MBP-cbbL-R. A 1.6 kb DNA fragment of the *cso* operon was amplified using primer pair cbbL-MBP-F and cbbL-MBP-R. Above two DNA fragments were fused using overlapping PCR. The product was digested with BamHI and BstZ17I, then cloned into pZS13S-23641-CbbL$_{6xHis}$ that was treated with the same restriction enzymes. The product is plasmid pZS-23641-MBP-Cbbl(C)6xHis.

**pZS-23641-CbbL-mCherry.** The mCherry gene was amplified using primer pair cbbL-mCherry-P1 and cbbL-mCherry-P2. A 0.8 kb fragment that contains the *cbbS* and the 5' of the *csoS2* genes was amplified using primer pair cbbL-mCherry-P3 and cbbL-mCherry-P4 from pZS-23641. The two PCR products were fused by overlapping PCR. The resulting DNA was digested with SalI and NheI, then cloned into pZS-23641 that was treated with the same restriction enzymes to yield pZS-23641-CbbL-mCherry.

**pZS-23641-CbbL(C)-mCherry.** To remove the CbbL(N), primer pair cbbL-Ndeletion-F and cbbL-Ndeletion-R was used to amplify plasmid pZS-23641-CbbL-mCherry by reverse PCR. The PCR product was directly ligated to form pZS-23641-CbbL(C)-mCherry.

**pZS-CsoS2(N6xHis).** The *csoS2* gene was amplified using primer pair csoS2-F and csoS2-R, which also added DNA sequence encoding 6xHis to the 5' of the *csoS2* gene. The PCR product was cloned into the SalI and HindIII sites of pZS13 to yield pZS-CsoS2(N6xHis).

**pZA-CbbL.** A 1.4 kb DNA fragment that encodes the CbbL of *H. neapolitanus* was amplified using primer pair cbbL-forward and cbbL-reverse. PCR product was cloned between the BamHI and HindIII sites on pZA33 to form pZA-CbbL.

**pET-CbbS.** A 0.33 kb DNA fragment encoding CbbS was amplified using primer pairs cbbS-forward and cbbS-reverse. The PCR product was cloned between the NdeI and XhoI sites on pET30b to form pET-CbbS.

**pZA-CbbLS.** A 2.0 kb DNA fragment that encodes of CbbL and CbbS was excised from pZS-23641 using restriction enzymes BamHI and NheI, then ligated into pZA33 that was cut with BamHI and AvrII to form pZA-CbbLS.

**pZA-CbbL(N)-mCherry6xHis.** The N domain of CbbL was amplified using primer pair cbbL-forward and cbbL(N)-mCherry from plasmid pZS-23641. The mCherry gene was amplified using primer pair mCherry-forward and mCherry-reverse. Above two PCR products were fused by overlapping PCR. The resulting DNA fragment was treated with BamHI and HindIII, then ligated into pZA33 that was digested by the same restriction enzymes to yield pZA-CbbL(N)-mCherry6xHis.

**pZS-23641-CsoS41.** A 1.6 kb DNA fragment that encodes the major shell proteins, including CsoS4A, CsoS4B, CsoS1C, CsoS1A and CsoS1B and CsoS1D, was amplified from pZS-23641 using primer pair cso41-forward and cso41-reverse from pZS-23641. The PCR product was directly ligated into pZS13 vector that was digested with SalI and HindIII to yield pZS-23641-CsoS41.

**pZA-cbbL(N)6xHis.** The DNA fragment that encodes the N domain of CbbL was first cloned into vector pET30b to construct an intermediate plasmid, which was used as the template for the amplification of CbbL(N)6xHis using primer pair cbbL(N)-forward and cbbL(N)-reverse. PCR products were cloned between BamHI and HindIII sites on pZA33 to form pZA-cbbL(N)6xHis.

**pET-csoS plasmids.** Each of the shell protein, including 1A, 1B, 1C, 1D, 4A and 4B was amplified using corresponding primers that are shown below. Each PCR product was digested by NdeI and XhoI, then ligated into pET30b that was treated with the same restriction enzymes to yield corresponding plasmid.
*csoS1A*, csoS1A-forward and csoS1A-reverse;
*csoS1B*, csoS1B-forward and csoS1B-reverse;
*csoS1C*, csoS1C-forward and csoS1C-reverse;
*csoS1D*, csoS1D-forward and csoS1D-reverse;

*csoS4A*, csoS4A-forward and csoS4A-reverse;
*csoS4B*, csoS4B-forward and csoS4B-reverse.

**pZA-cbbL(1-n)-MBP6xHis plasmids.** DNA fragments that encode varied segments of CbbL(N) were amplified from pZS-23641 using cbbL-forward primer and one of the following reverse primers. The MBP-encoding DNA was amplified from plasmid pMAL using primer pair MBP-forward and MBP-reverse. The MBP PCR product was then fused with each of the CbbL(N) segments, respectively. The resulting products were digested with BamHI and HindIII, then ligated into pZA33 that was treated with the same enzymes.
*cbbL*(1-20), cbbL20-reverse;
*cbbL*(1-73), cbbL73-reverse;
*cbbL*(1-100), cbbL100-reverse;
*cbbL*(1-122), cbbL122-reverse;
*cbbL*(1-136), cbbL136-reverse.

**pZA-cbbL(1-136)-MBP.** The DNA fragment of *cbbL*(1-136)-MBP was amplified using primer pair cbbL-forward and cbbL136-MBPnoHis from plasmid pZA-cbbL(1-136)-MBP6xHis. The PCR product was digested with BamHI and PspXI, then ligated into pZA33 that was treated with the same enzymes to form pZA-cbbL(1-136)-MBP.

**pET-csoS1C6xHis.** The *csoS1C* gene together with its native RBS was amplified using primer pair csoS1C6xHis-F and csoS1C6xHis-R from plasmid pZS-23641. The PCR product was cloned between the XbaI and XhoI sites on pET30b to yield pET-csoS1C6xHis.

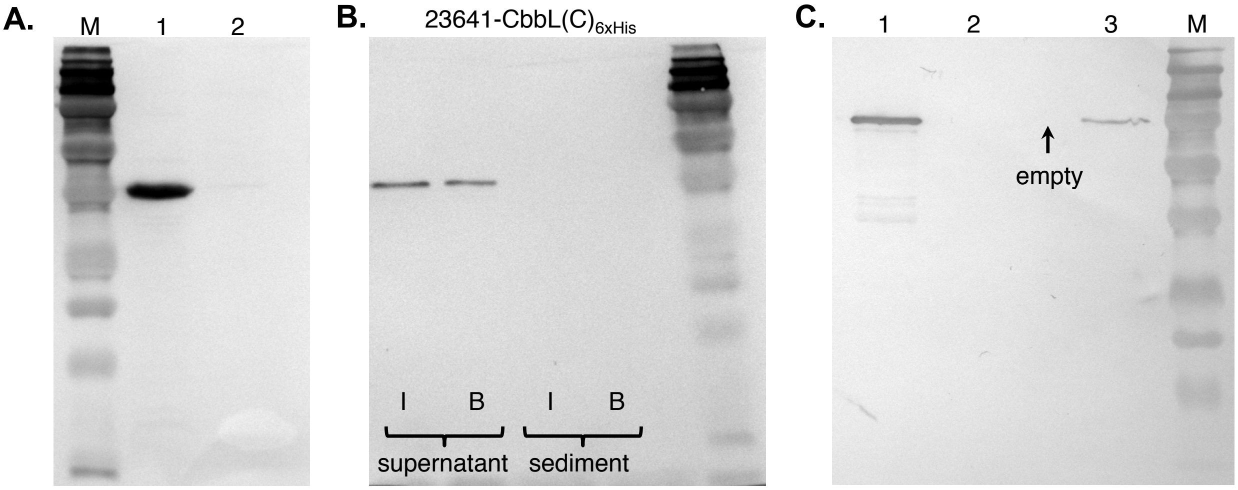


**Figure S1. Solubility study of CbbL(C) and MBP-CbbL(C) using western blot.** (A) Solubility of CbbL(C) expressed from *E. coli* transformed with pZS-23641-CbbL(C)$_{6xHis}$ (Original image, Figure S11). lane 1, total fraction of cell lysate; lane 2, soluble fraction of cell lysate; (B) Immunoprecipitation of purified carboxysome from *E. coli* cells transformed with pZS-23641-CbbL(C)$_{6xHis}$(Original image, Figure S12). I, sample of intact carboxysome; B, sample of broken carboxysome; (C) Solubility of MBP-CbbL(C) expressed from *E. coli* transformed with pZS-23641-MBP-CbbL(C)$_{6xHis}$ (Original image, Figure S13). lane 1, total fraction of cell lysate; lane 2, purified carboxysome from *E. coli* cells transformed with pZS-23641-MBP-CbbL(C)$_{6xHis}$.; lane 3, soluble fraction of cell lysate. All experiments used the 6xHis tag monoclonal antibody to detect the tagged protein. In all images, M is molecular weight markers.

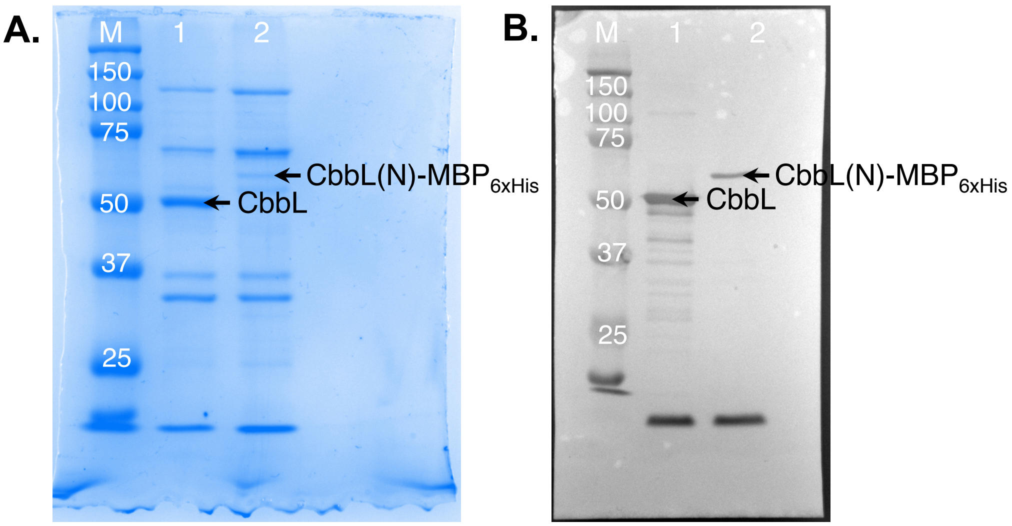


**Figure S2. Analysis of purified carboxysome 23641-CbbL(N)-MBP$_{6xHis}$** (A) SDS-PAGE of purified carboxysomes (Original image, Figure S14). M, protein size markers; lane 1, from cells transformed with plasmid pZS-23641-CbbL$_{6xHis}$; lane 2, from cells transformed with pZS-23641-CbbL(N)-MBP$_{6xHis}$; (B) Western blot of purified carboxysomes (Original image, Figure S15). M, protein size markers; lane 1, from cells transformed with plasmid pZS-23641-CbbL$_{6xHis}$; lane 2, from cells transformed with pZS-23641-CbbL(N)-MBP$_{6xHis}$;

**A.**

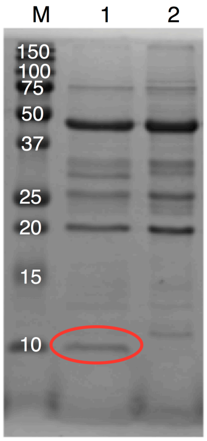


**B.** CsoS1A

M**ADVTGIALG MIETRGLVPA IEAADAMTKA AEVRLVGRQF VGGGYVTVLV**
**RGETGAVNAA VR**<u>AGADACER</u> **VGDGLVAAHI IARVHSEVEN ILPKAPQA**

**C.** CsoS1C

M**AAVTGIALG MIETRGLVPA IEAADAMTKA AEVRLVGRQF VGGGYVTVLV**
**RGETGAVNAA VR**<u>AGADACER</u> **VGDGLVAAHI IARVHSEVEN ILPKAPEA**

**Figure S3**. **Identification of shell proteins that interact with CbbL(N).** (A) SDS-PAGE of pull-down experiments (Original image, Figure S16). M, molecular size markers; lane 1. Cell lysate of *E. coli* that co-expressed CbbL(N)-mCherry-6xHis and shell proteins of carboxysome; lane 2. Cell lysate of *E. coli* that expressed CbbL(N)-mCherry-6xHis. Protein band that is circled in red in lane 1 was subjected in-gel digestion and proteomics analysis using mass spectrometry; (B) and (C) Sequence coverage of CsoS1A and CsoS1C in proteomics analysis of excised band from (A). Sequence that was detected is in bold. Sequence that was not detected is underlined.

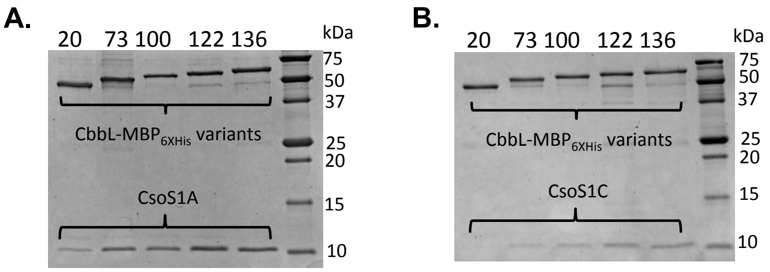


**Figure S4. Pull-down experiments between truncated CbbL(N) and major shell proteins.** (A) CsoS1A (Original image, Figure S17); (B) CsoS1C (Original image, Figure S18).

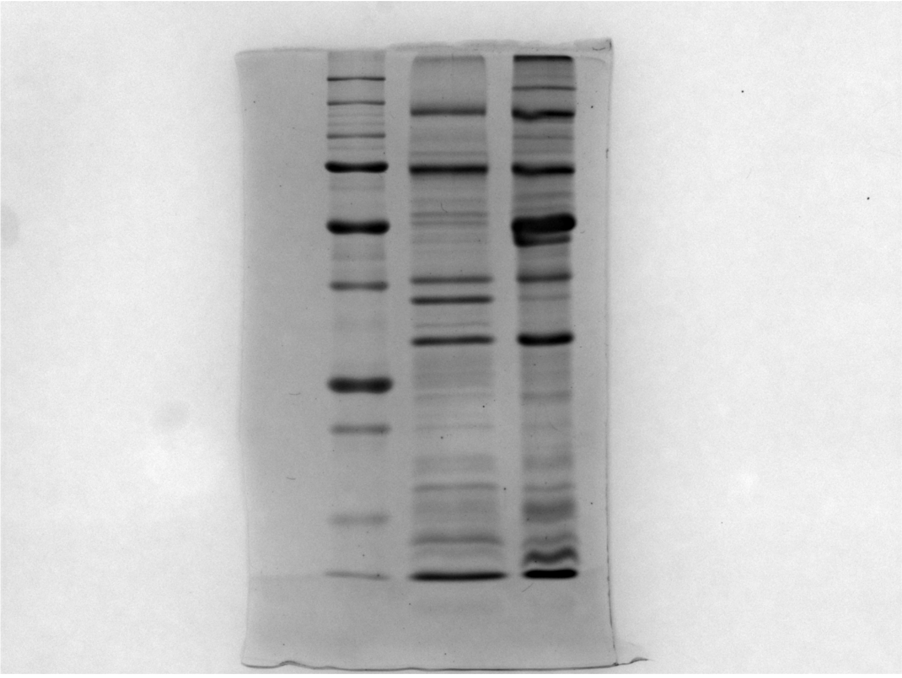

**Figure S5. Original SDS-PAGE image of Figure 2A.**

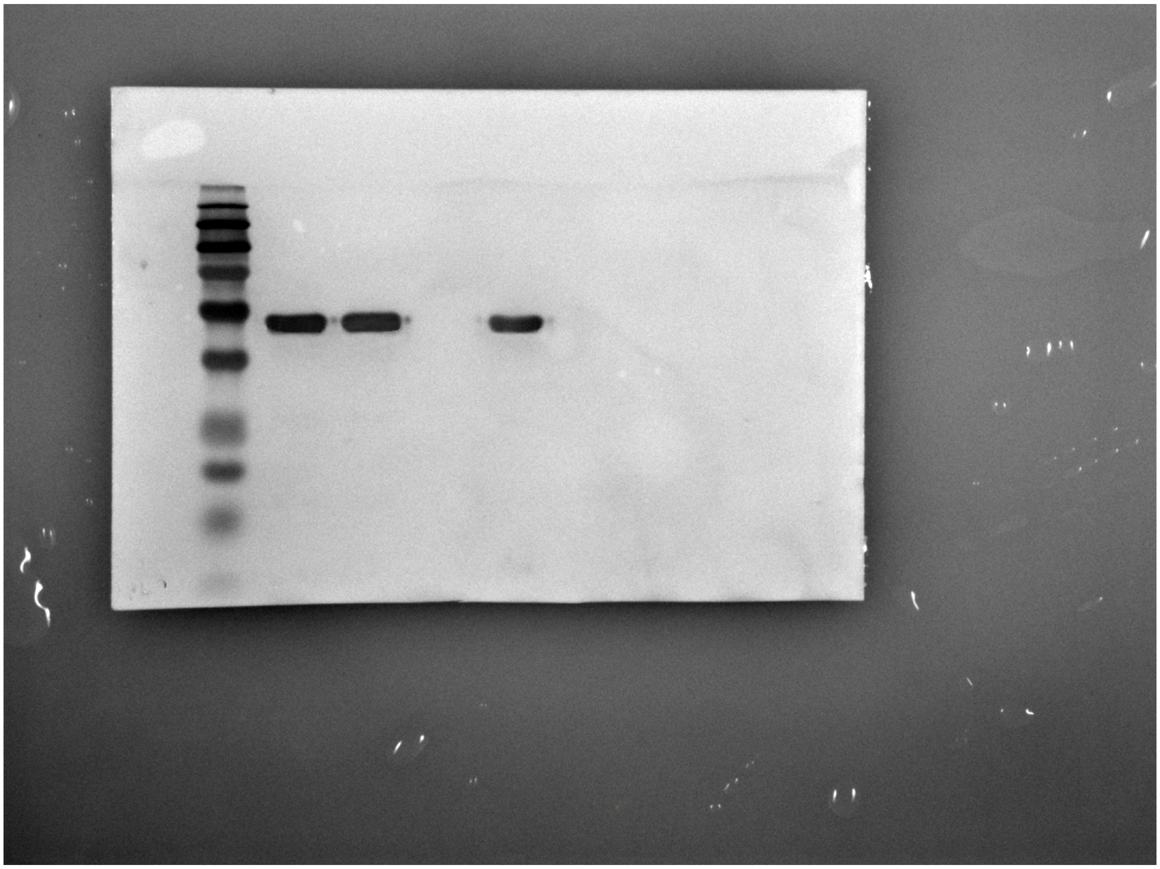

**Figure S6. Original western blot image of Figure 2B.**

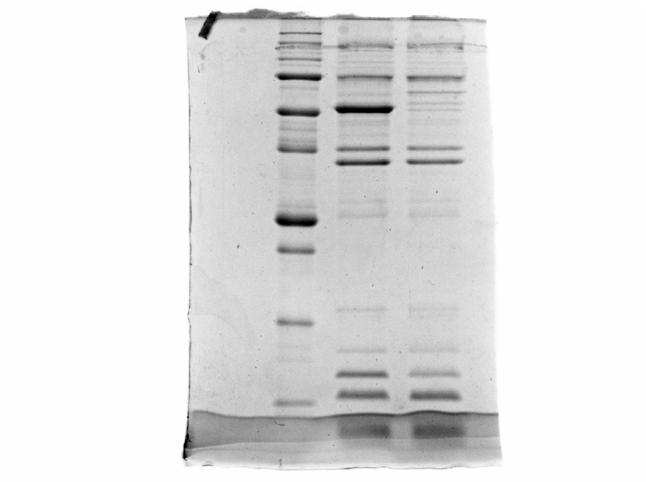

**Figure S7. Original SDS-PAGE image of Figure 3A.**

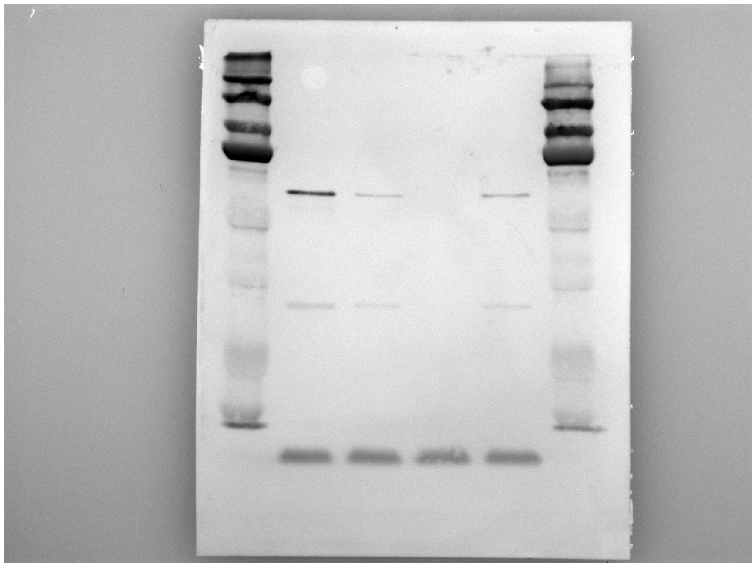

**Figure S8. Original western blot image of Figure 3B.**

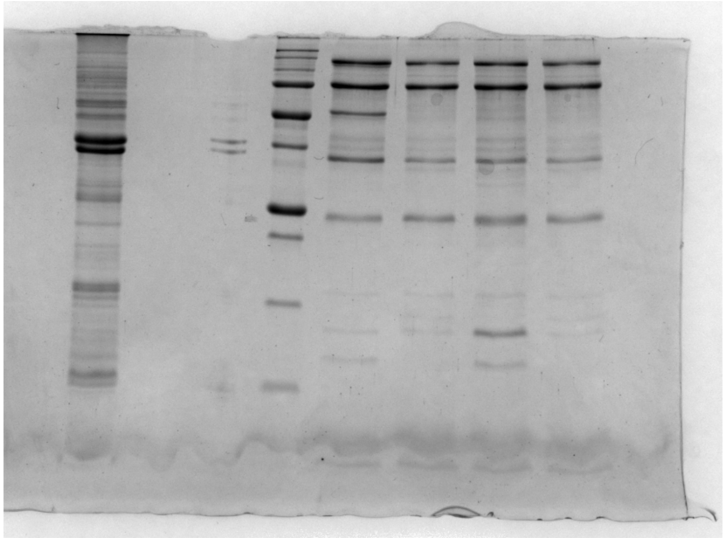

**Figure S9. Original SDS-PAGE image of Figure 4.**

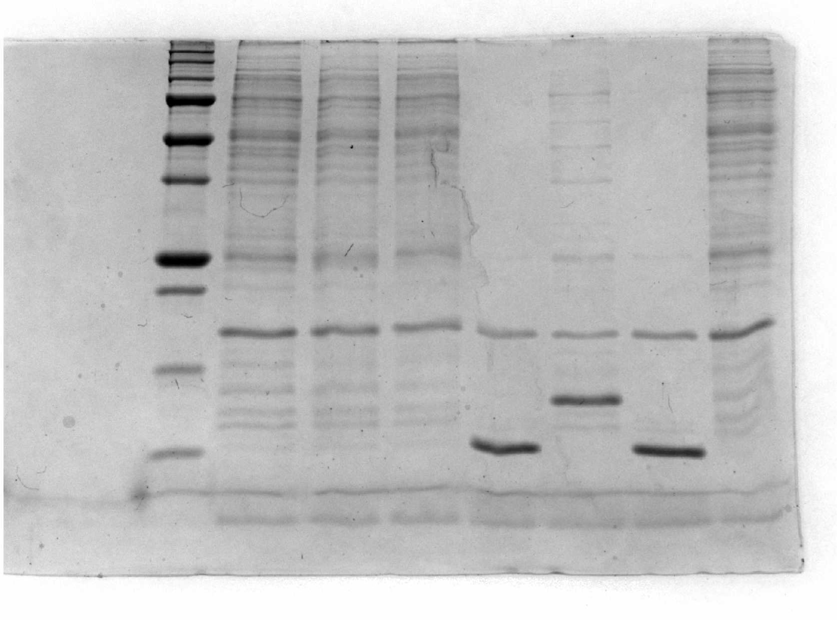

**Figure S10. Original SDS-PAGE image of Figure 5.**

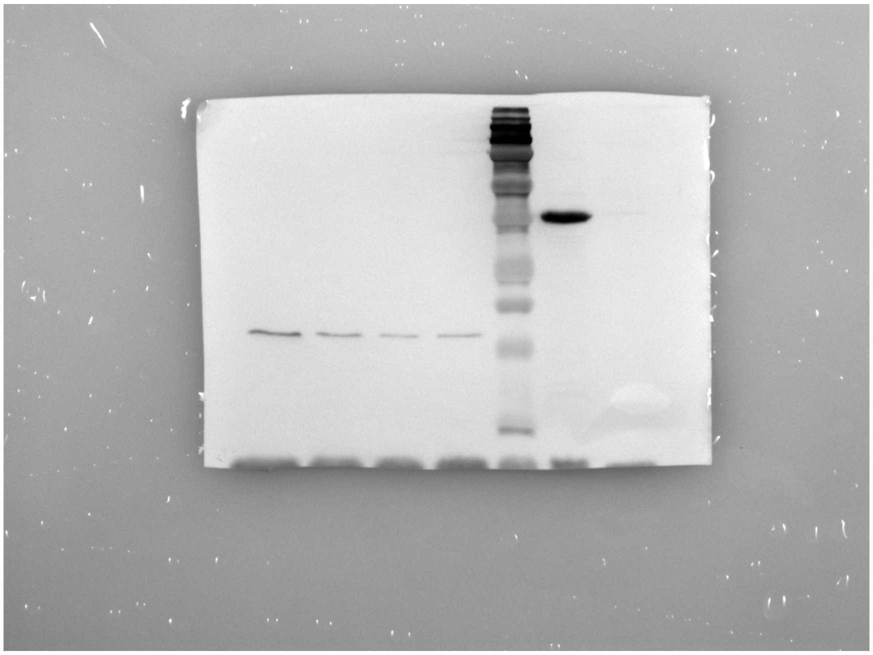

**Figure S11. Original western blot image of Figure S1A.**

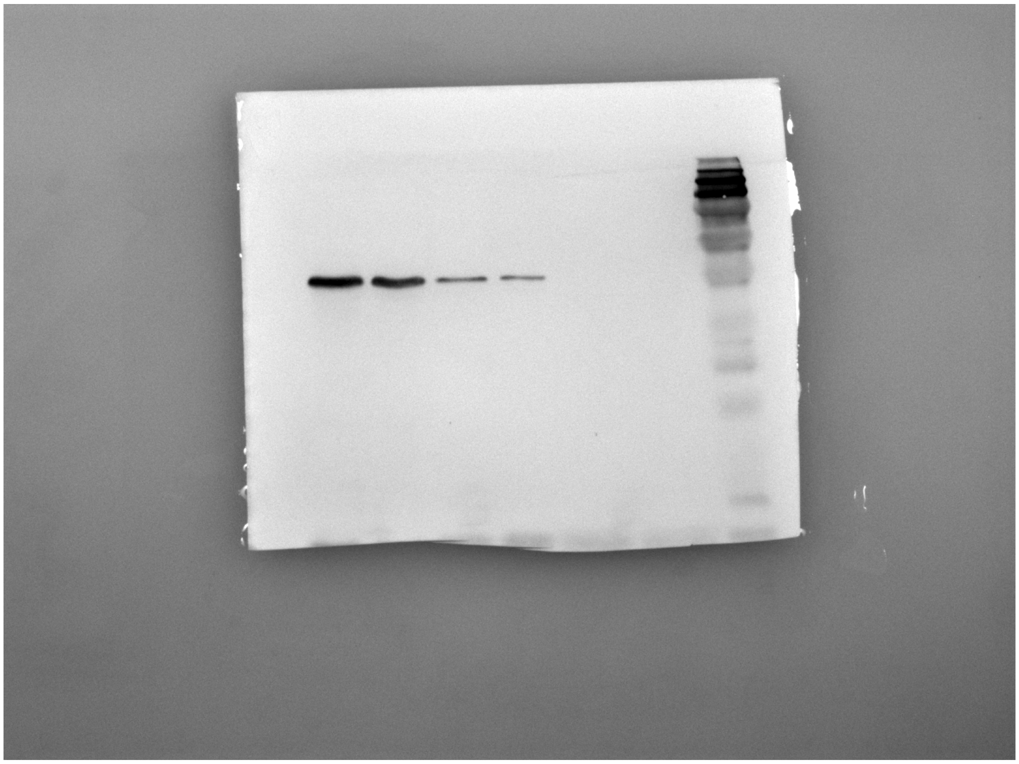

**Figure S12. Original western blot image of Figure S1B.**

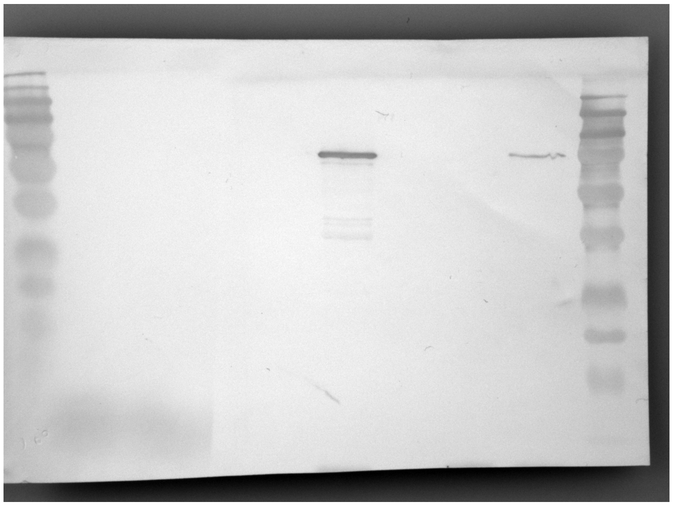

**Figure S13. Original western blot image of Figure S1C.**

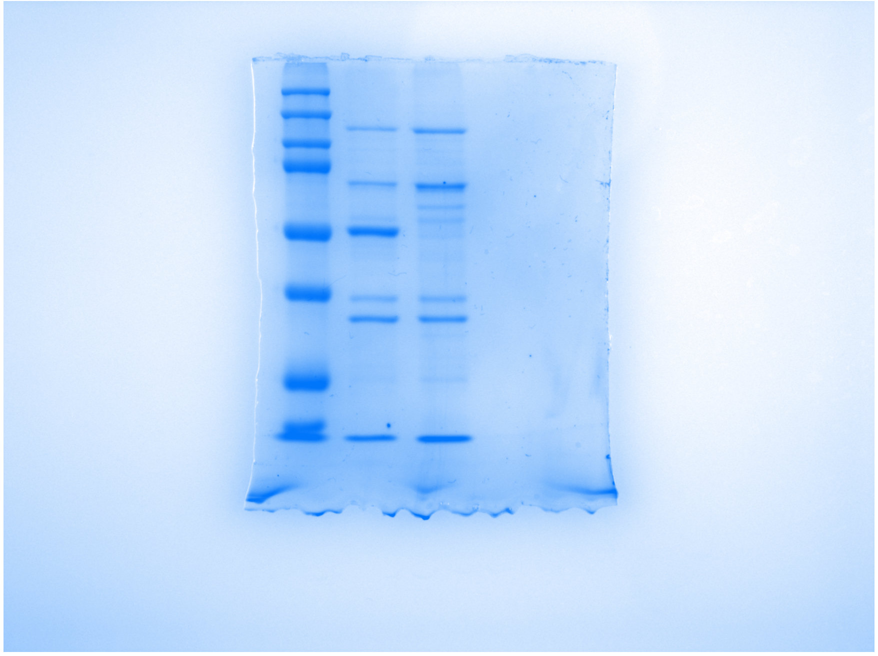

**Figure S14. Original SDS-PAGE image of Figure S2A**.

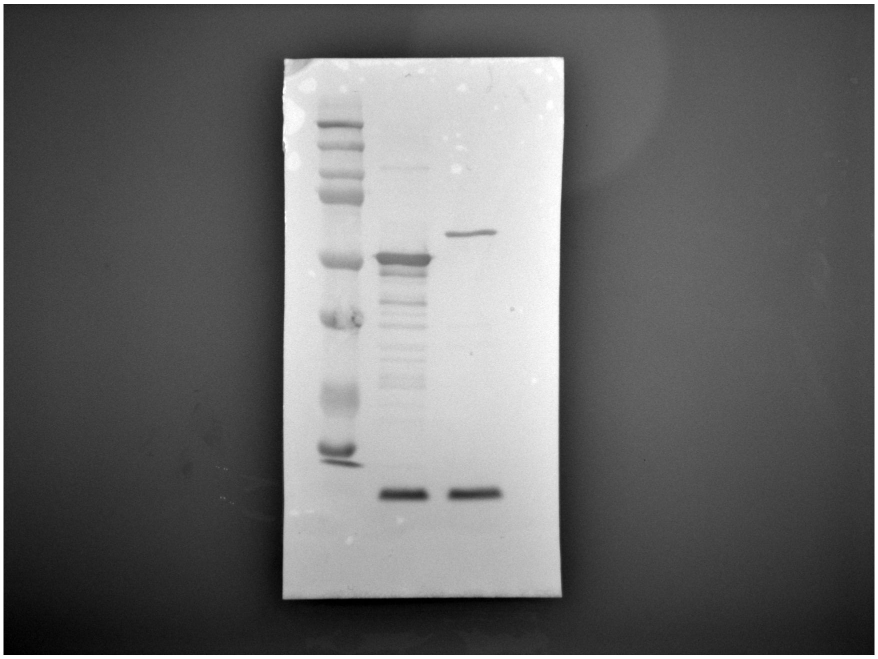

**Figure S15. Original SDS-PAGE image of Figure S2B.**

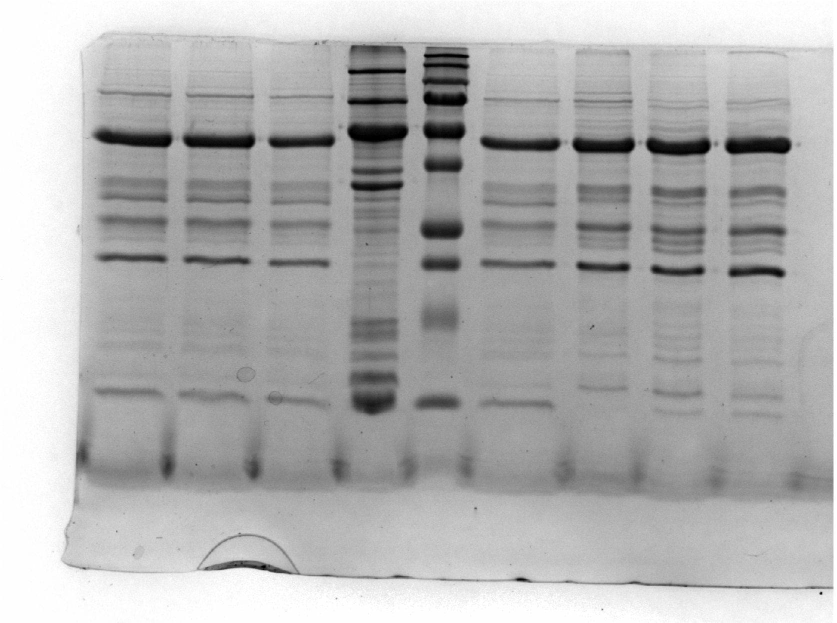

**Figure S16. Original SDS-PAGE image of Figure S3**.

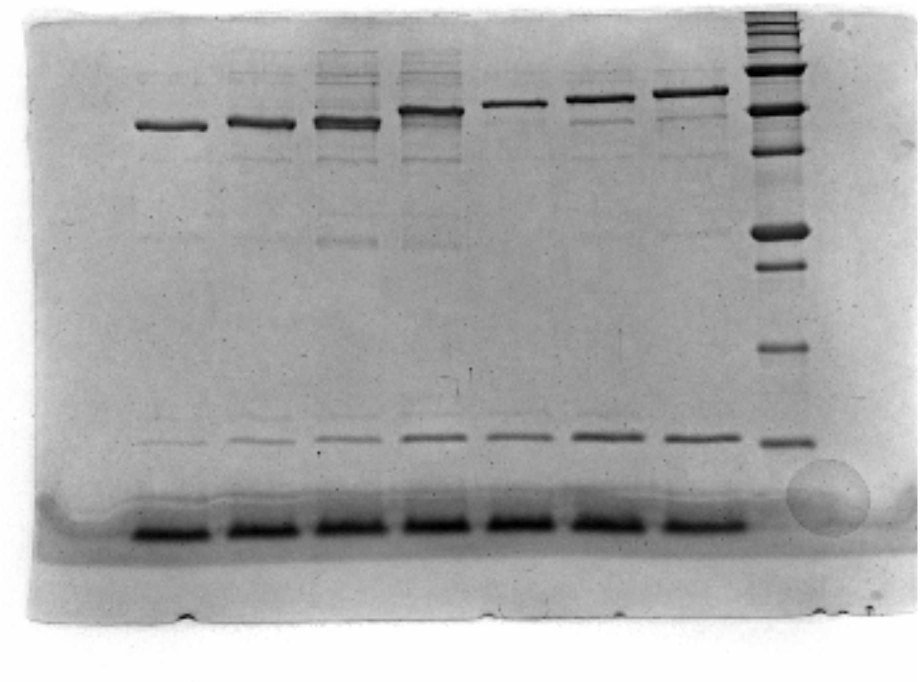

**Figure S17. Original SDS-PAGE image of Figure S4A**.

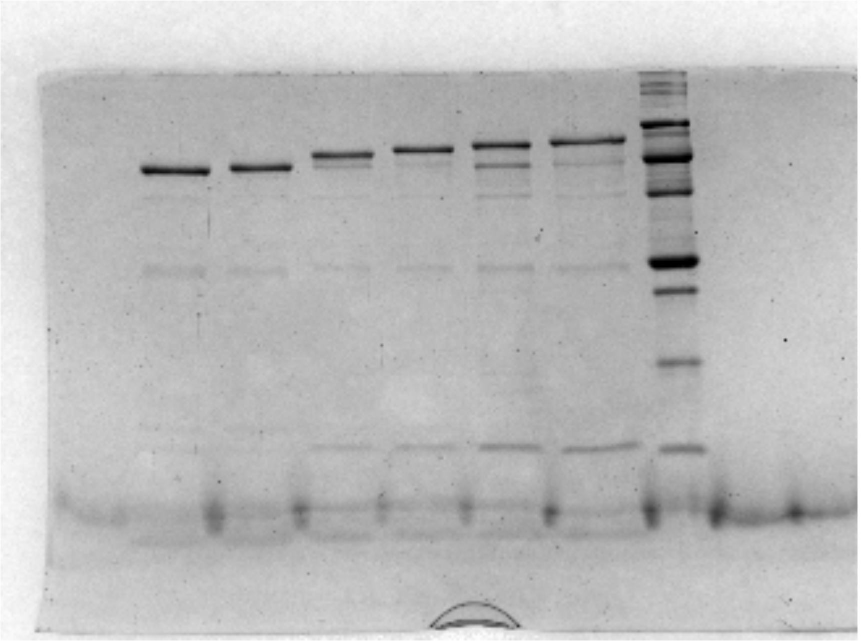

**Figure S18. Original SDS-PAGE image of Figure S4B**.
